# An offline deep learning model for bovine muzzle identification using the variance of the laplacian, YOLOv8, EfficientNetV2-S, and ArcFace

**DOI:** 10.1186/s12917-026-05948-0

**Published:** 2026-10-19

**Authors:** Ahlam G. Khalifa, Ahmed S. Abdultawab

**Affiliations:** 1 Department of Forensic Medicine and Toxicology, Faculty of Veterinary Medicine, Beni-Suef University, Beni-Suef, 62511 Egypt; 2 Faculty of Veterinary Medicine, Beni-Suef University, Beni-Suef, 62511 Egypt

**Keywords:** Identification, Biometric, Efficient NetV2-S, ArcFace, Offline

## Abstract

Precise animal identification underpins livestock management, disease control, and traceability. Traditional methods such as tattoos and ear tags can fade or be lost, motivating a shift toward biometric-based systems. This study proposed an offline Python-based deep learning biometric identification model for bovine using muzzle images. YOLOv8 detected and cropped the muzzle region, EfficientNetV2-S architecture was integrated with ArcFace (angular margin loss) for muzzle pattern extraction and matching. The model was evaluated under two conditions: a controlled dataset (Kaggle) and a preliminary, small-scale field dataset using smartphone-acquired images from smallholder farmers. The model was validated using the Kaggle dataset and tested on both Kaggle and field-collected muzzle images. Model performance was evaluated using the cosine similarity score, processing time, F1 score, recall, accuracy, and precision. The model showed shorter processing times and higher recognition performance on the Kaggle images than on the field images. A domain gap was observed between Kaggle dataset and field dataset. The current model is a controlled prototype with preliminary field testing for offline muzzle recognition; however, it has not yet met pre-defined field readiness measures. Overall, the proposed offline muzzle biometric model provides a non-invasive prototype for bovine identification under the tested conditions.

**Supplementary Information:**

The online version contains supplementary material available at https://doi.org/10.1186/s12917-026-05948-0.

## Introduction

 Traceability of animal products increases consumer confidence and food safety particularly for meeting international trade standards [1, 2]. The origin of the meat products is verified by DNA-based identification methods, preventing adulteration [3, 4]. In biological research, reliable animal identification helps ensure reproducibility and integrity of data in experimental research, especially long-term studies by linking each result and observation to its correct animal, eliminating systematic errors arising from misidentification [5]. When data from misidentified animals are aggregated, they can lead to false-positive or false-negative evaluations skewing perceived treatment outcomes [6]. Precision feeding systems based on ear tags only for animal identification showed limited cow-visit identification performance in a specific live-stream ear-tag reading experiment [7].

In genetic mapping, accurate animal identification ensures that genetic data are correctly attributed to individuals or species [8], facilitates marker-assisted selection (MAS) for desirable features in aquaculture [9],and supports linkage maps for quantitative trait loci (QTL) and identification of single-nucleotide polymorphisms [10]. Also, facilitates genetic research in species with limited genomic data [11].

Animal identification programs are important during disease outbreaks such as swine flu, foot-and-mouth disease (FMD), and bovine spongiform encephalopathy (BSE). These programs facilitate quick and accurate tracking of infected animals to control disease spread [12]. Concerning vaccination, accurate animal identification ensures that animals receive proper vaccinations [13]. In livestock farming, identification systems such as biometric methods and electronic chips prevent theft and establish ownership and, in livestock commerce, may reduce fraudulent substitutions and false insurance claims [1].

Traditional livestock identification methods have been widely used for decades, but they do not depend on distinctive and immutable features of the animals, making them susceptible to theft and identity swapping [14]. Some of them negatively impact animal welfare because procedures such as ear notching can be painful [15]. Ear tags can fall off, microchips can malfunction, and tattoos can fade, thus they are unsuitable for long-term accurate identification [16]. Tags lost during grazing may represent a complication for the vaccine efficacy studies [17]. The shortfalls in the conventional identification methods catalyzed a shift toward biometric-based systems [15].

Muzzle patterns are unique to every cow analogous to human fingerprints. Also, they are visible and distinctive trait thus they used as a dependable way for animal identification [18]. Muzzle-based identification is a highly accurate, cost-effective, non-invasive, durable, and versatile approach that bypasses the limitations of traditional identification methods [19].

Biometrics refers to technologies that measure and analyze unique physiological or behavioral characteristics of an individual for authentication and identification. Physiological traits in humans, such as fingerprints, iris patterns, and facial features, are increasingly used in smartphones and airports [20]. For animal muzzle biometrics, features have been extracted using histograms of multichannel local binary patterns [19]. The SIFT algorithm has also been used for feature extraction from muzzle images [21]. Retinal patterns have been analyzed using software integrated into the Optibrand OptiReader device [22]. YOLOv11 has also been used for cattle facial recognition [23]. Nose-print biometrics have also been investigated in dogs [24].

Despite the recent biometric methods of cattle identification, some limitations remain present. A previous study achieved 93.3% identification accuracy [25], but it used a small-scale dataset limiting its field application. The modern computer vision applications based on deep learning have often shown better performance than those relying on the traditional feature extraction techniques such as ORB and SIFT. Most livestock identification systems used ORB and SIFT [26]. Furthermore, there are restrictions on their real-time application. Pathak and Prakash [15] reported a fine-grained image-analysis system with 99.47% identification accuracy; however, the system is limited by its reliance on high-quality muzzle images, high-end cameras, strict head angles, and constant internet connectivity.

Recent studies identified the cattle based on deep-learning, in a frame work, Shojaeipour et al. [27] used YOLOv3 and SoftMax classification. Pathak and Prakash [28] published a YOLOv8-based approach but this approach was classification-based. Li et al. [29] proposed multiple deep-learning models for individual cattle identification but these models were not evaluated against unregistered cows. The present work based on YOLOv8 which is more recent than YOLOv3., moved from SoftMax classifier to ArcFace, which is superior to classification-based one., and integrated the EfficientNetV2-S architecture with ArcFace (angular margin loss) to enhance intra-class compactness and inter-class separation yielding a 512-dimensional embedding spaces for similarity matching. The evaluation not based only on embedding-based accuracy identification using cosine-similarity matching but also, estimated the threshold-based rejection rate of unregistered cows. The model was designed for offline operation in farm environments, with inference exported to ONNX.

## Materials and methods

### Hardware and software deployment

The model was deployed and tested on a machine running the Windows 11 operating system, powered by an 11th Gen Intel^®^ Core™ i9-11950 H CPU. The pipeline was built using ONNX Runtime v1.23.2. The model architecture was exported via ONNX opset 14.

*EfficientNetV2-S implementation-level recipe*: torchvision EfficientNetV2-S, ImageNet default pretrained weights, 224 × 224 input, ImageNet normalization, 1280-to-512 projection, BatchNorm1d, no dropout, five frozen-backbone epochs, AdamW, learning rate `3e-4`, weight decay `1e-4`, 50 epochs, batch 32, cosine warm restarts, augmentations, and Windows-safe zero-worker loading [30].

### Data acquisition

The data acquisition for this study relied on:

A) *Cow Muzzle Dataset sourced from Kaggle (Version 1)*, which supplied us with an extensive collection of biometric images [31]. A total of 1,300 images (160 cows, 8 ± 1images per cow) were partitioned: Enrollment/Gallery Set (820 images) constructed the reference templates for each cow (5 ± 1 images per cow), Validation Set (320 images), and Test Set (160 images) to assess the accuracy of the model. This dataset contains high-resolution muzzle images under varying conditions and angles and shows fine-grained texture details of the muzzle pattern (640 × 640 pixels in 3-channel RGB JPEG, frontal close-up views of cows). This variation may support robustness testing, but detection performance must be demonstrated empirically.
B) *Field Data*: the researchers constructed the field dataset by capturing the muzzle images from smallholder farmers in Beni-Suef Governorate using a smartphone equipped with a 64-MP camera. Each farmer owned an average of 7 cattle. The high-resolution frontal macro images (768 × 1024 pixels) were captured during daylight at a fixed distance of 15–20 cm from the muzzle without flash. Several shots, across two distinct sessions with one week interval, were acquired per animal to include variations in angle and moisture distribution. These images were locked and never accessed during model validation. A total of 400 images (30 cows and 22 buffaloes) were partitioned: Enrollment/Gallery Set constructed the reference templates for each animal (6 ± 1 image per animal) and was selected from the first session (348 images), and Test Set was taken from the second session (52 images). Theses raw images were enrolled and tested by the model without any manual editing or pre-processing to represent real-world conditions. The model was evaluated in closed-set identification and open-set recognition. Closed-set identification assuming that the probe belongs to one of the enrolled animals in the gallery and the model decided which known identity. In open-set recognition, probes belong to unenrolled animals and the model performance was assessed using positive rejection rate and false acceptance rate. Evaluation protocol for the closed-set and the open-set rejection of unregistered cattle was shown in Table 1.

**Table 1:** Evaluation protocol for the closed-set and the open-set rejection of unregistered cattle

	closed-set identification	open-set rejection of unregistered cattle
Gallery size	820 Kaggle + 348 field images	820 Kaggle + 348 field images
Gallery identities	212 (registered cattle)	212 (registered cattle)
Mated probes	212	212
Un registered probes	0	80 (Kaggle images)
Search type	1:N classification	1:N classification

### Blur detection step

**Fig. 1:**
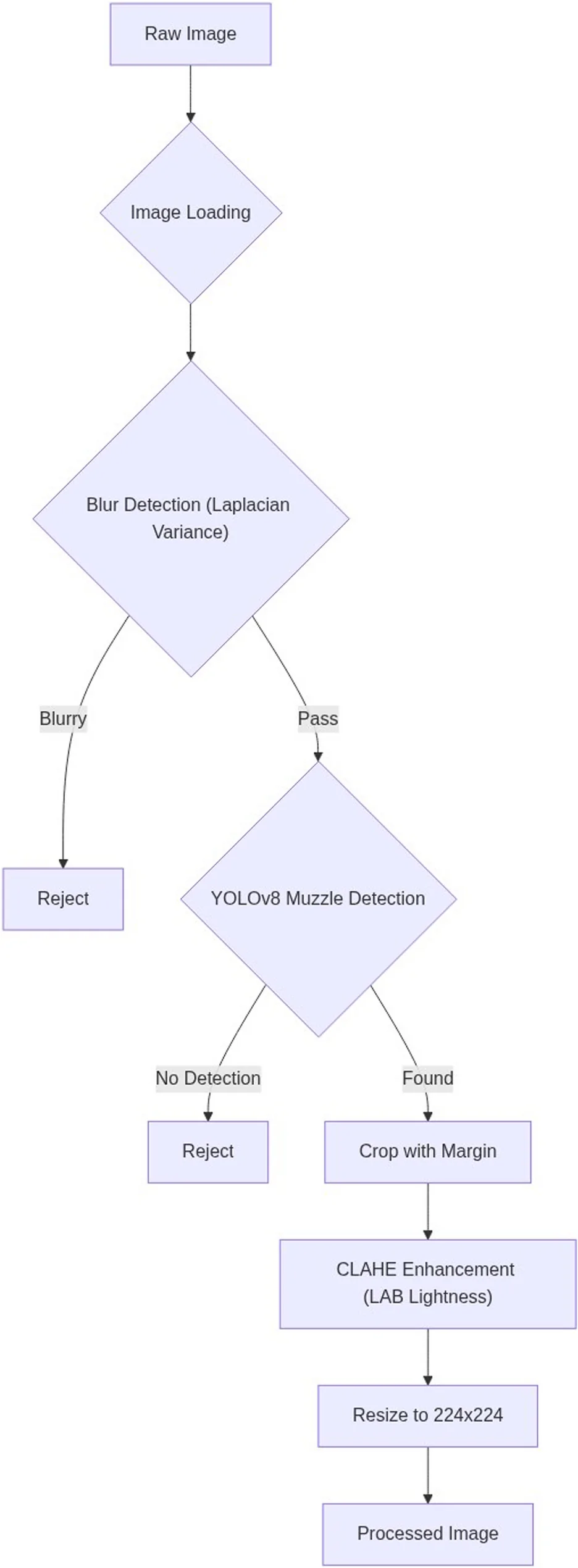
Muzzle-image processing pipeline

Input images were subjected to blur detection as a part of the pre-extraction pipeline. The variance of the Laplacian method was used for this pipeline [32]. An empirical threshold value was selected to categorize the muzzle images as either sharp or blurred. Images with blur scores below the threshold (A blur rejection threshold of *τ*blur = 4.0) were rejected to enhance the accuracy and precision of the model as shown in Fig. 1.

### Muzzle detection

The muzzle region in each input image was detected, localized and cropped using YOLOv8 (You Only Look Once) algorithm [33]. The detector configuration was reported in (Table 2) [34]. The local contrast was enhanced using CLAHE Contrast Limited Adaptive Histogram Equalization (CLAHE), thereby enhancing local contrast and fine-detail visibility [35]. The exact CLAHE parameters were contrast threshold: 2.5, grid size: 8 × 8, and L^∗^ channel).

**Table 2:** Validation performance results of the cattle muzzle identification model on Kaggle database

Data set	Accuracy (%)	Recall (%)	Precision (%)	F1-score (%)	FRR (%)	Average cosine similarity (%),95%CI	Average matching time (ms), 95%CI	Average extraction time (ms), 95%CI
kaggle database	90.3	90.3	95.37	92.77	9.78	88.42 (85.69,91.16)	6.9 (6.68,7.20)	36.91 (35.49,38.33)

*95%CI* 95% confidence interval, *FRR* false rejection rate

### Feature extraction

Feature extraction is an important step in biometric bovine identification by converting the muzzle image pattern into a numerical vector. These features allow the system to accurately identify and differentiate between animals. For the feature extraction stage, we use pre-trained EfficientNetV2-S as the default backbone CNN, combined with the ArcFace loss function with angular margin (0.5 rad) [36]. In this model, EfficientNetV2-S was employed because of its reported parameter/training efficiency [37]. The network compressed the images into a low-dimensional embedding within the space.

ArcFace was added to the model to boost the accuracy of muzzle identification. In ArcFace, we applied a scaling factor of 30 to increase the separation between classes during metric learning. Introducing angular margin of *m* = 0.50 and a hypersphere scale of *s* = 30.0 between classes encourages the intra-class feature to be closer together, while increasing the inter-class feature distances [38] so muzzle images of the same animal are gathered more closely together, while images from different animals are separated further making these features more appropriate for identification. In our model we apply a top-1 vs. top-2 angular separation threshold = 0.03 rad (≈ 1.72∘) to reject false positive identification. The resulting embedding was mapped into a 512-dimensional space and normalized via L2 normalization, placing each L2-normalized embedding on the unit hypersphere.

For the biometric identification, EfficientNetV2-S extracted the visual features from the cropped muzzle then encoded them as a learned 512-dimensional latent embedding via ArcFace [39]. These embeddings capture textural and structural characteristics of the muzzle.

### Evaluation methodology

NIST biometric evaluation standards were followed to conduct the comprehensive operating-point analysis with prespecified selection rules on validation set [40].

#### Matching strategy

**Fig. 2:**
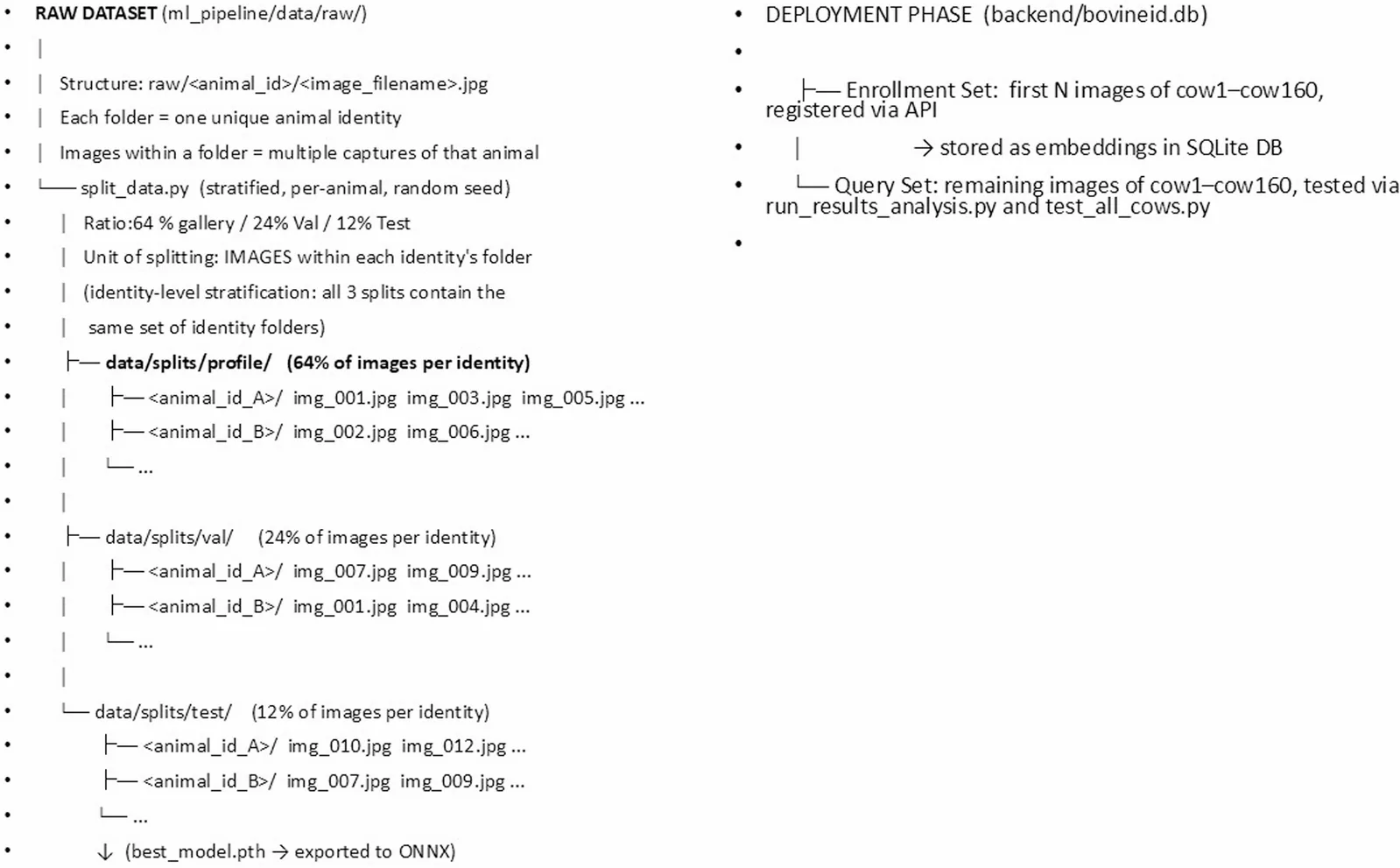
Data partition and deployment

The matching was based on templet matching. Figure 2 showed the data partition and deployment of the enrolled and tested muzzle images.

- *Enrollment*: Multiple images (5 ±2) per known animal were passed through the model, and their embeddings were extracted and normalized to form the template that represents the animal''s identity.
- *Query Processing*: The new unknow muzzle image underwent the same preprocessing and extraction pipeline as the enrollment images to form a query embedding which was matched against the stored templates of the enrolled known animals.
- *Similarity Computation*: The normalized feature vector was compared using cosine similarity, yielding a similarity score. The top-ranked candidate was accepted if its similarity score was above the similarity threshold (0.70). If cosine similarity ≥0.70 but angular separation<0.03 rad, the unknown image is rejected.

#### Evaluation metrics

For Closed-set identification: the model’s performance was evaluated during the validation and testing stages using average cosine similarity (%), average matching time (ms), average extraction time (ms), accuracy ((TP + TN) / total count), recall (TP / (TP + FN)), precision (TP / (TP + FP)), and F1-score (2 * (Precision * Recall) / (Precision + Recall)).

For open-set recognition: we evaluated the positive rejection rate (True negative / Total impostor attempts *100) and false acceptance rate (FP / Total impostor attempts *100).

### Design rationale

*CLAHE preprocessing*: CLAHE boosts the contrast and limits the noise amplification making texture patterns picking easier for the network. Recent work combining CLAHE with YOLOv8 for biometric-related detection reported improvement in precision and recall over using raw images [41].

*YOLOv8*: The muzzle region in each input image was detected, localized and cropped using YOLOv8 algorithm [33]. YOLOv8 is the most advanced model in the YOLO lineup with architectural improvements (better backbone, neck and loss functions) [42].

*EfficientNetV2-S*: pre-trained EfficientNetV2-S was used as the feature-extraction default backbone CNN. the modern identification systems used EfficientNetV2-S due to their high computational efficiency and representational power.

*ArcFace*: added to the model to boost the accuracy of muzzle identification. Softmax loss optimized for linear separability, which is inadequate for fine-grained open-set recognition. ArcFace integrated with the EfficientNetV2-S architecture to enhance intra-class compactness and inter-class separation making the open-set detection much more reliable.

*Angular rejection rule*: If cosine similarity ≥ 0.70 but angular separation < 0.03 rad, the unknown image is rejected. Enforcing severe mathematical conformity to the learned embedding space by imposing an angular rejection rule drastically reduced false acceptance rate.

## Results

### Operating-point analysis

**Fig. 3:**
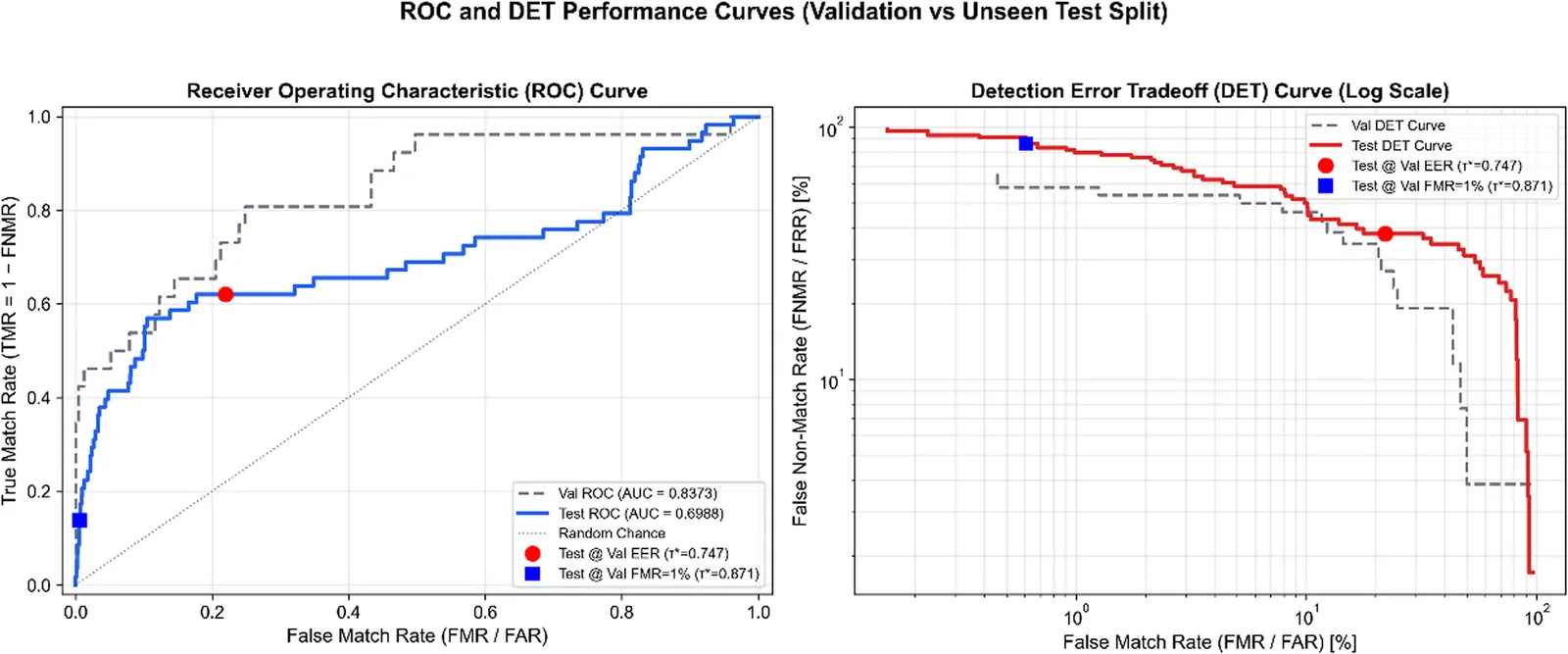
ROC and DET performance curves

ROC curve and DET curve were used to analyze performance, yielding an Equal Error Rate of 23.95%. ROC and DET performance curves showing True Match Rate (TMR) vs. False Match Rate (FMR). The validation set achieved an AUC of 0.8373. at the Equal Error Rate (EER) threshold, TMR = 0.747; at the 1% FMR threshold, TMR = 0.871 Fig. 3.

### Threshold selection

The threshold was selected at τ∗=0.70 (70% cosine similarity), joined with a secondary ArcFace angular separation margin of Δθ ≥ 0.03 rad to reject false positive identification. To formalize this operating point, three reference operating points were derived on the validation split:

- Equal Error Rate — EER Rule: Threshold τE∗ER=0.7469 selected where FNMR = FMR on val (Val EER = 23.95%).
- High-Security Rule: Threshold τF∗MR1=0.8708 selected where FMR ≤0.01 on val.
- High-Convenience Rule: Threshold τF∗NMR5=0.6811 selected where FNMR ≤0.05 on val.

### Enrollment and identification results displayed by the proposed model

**Fig. 4:**
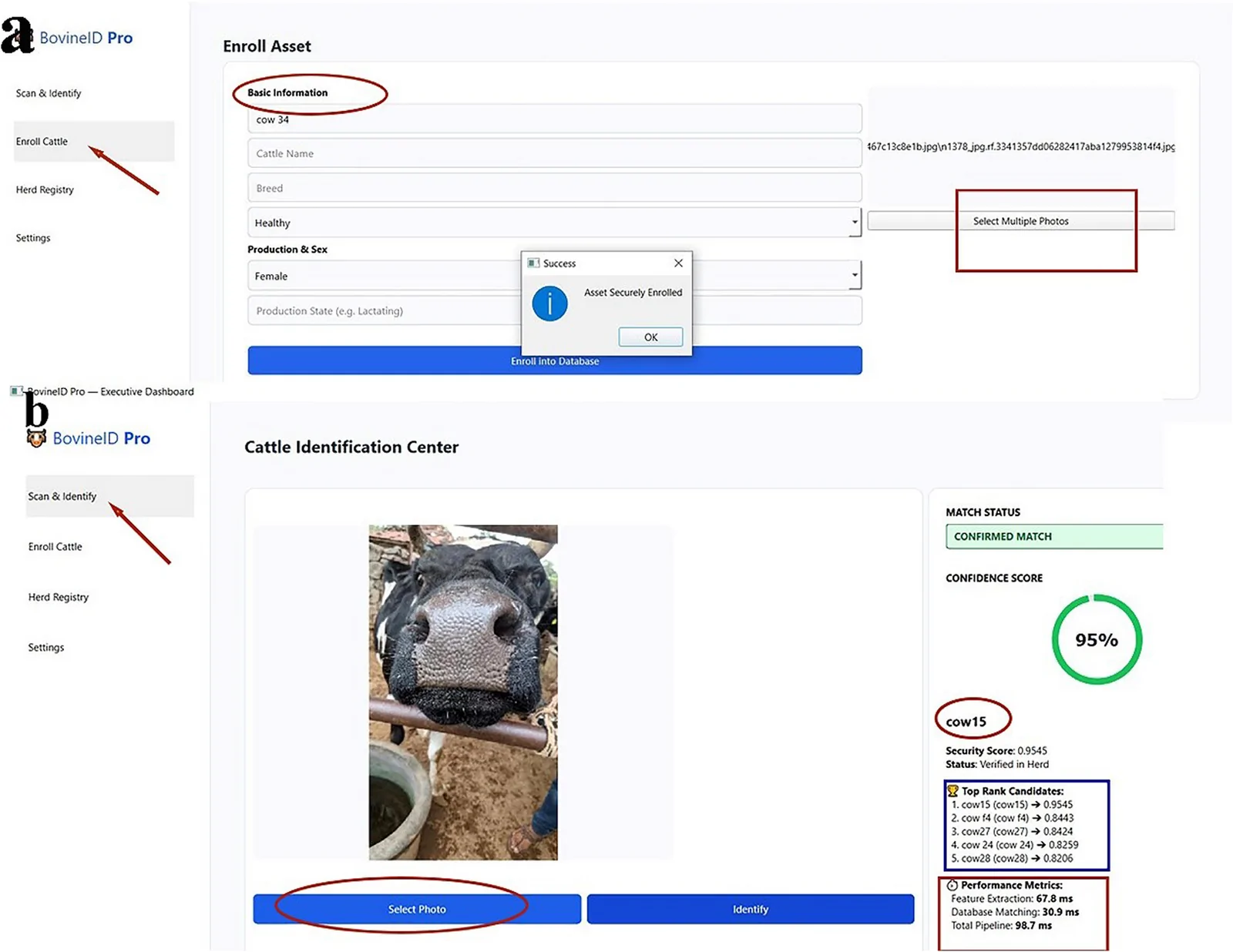
The user interface of the muzzle identification system. The system allows users to upload the Template for herd registry (**a**) and scan the query muzzle then view the identification results, including the cattle number, cosine similarity, extraction time, and matching time. In the illustrated example, the query muzzle was successfully identified with a cosine similarity of 95% (**b**)

The system allows users to upload a muzzle image for herd registry by enrolling a cow, entering its basic information, and uploading multiple muzzle images. Successful enrollment is indicated by a confirmation message as shown in Fig. 4a. For unknown muzzle identification, the user uses the Scan & Identify command, selects a photograph, and initiates identification. After feature extraction and similarity matching, the model displays the predicted cattle number, cosine similarity, extraction time, and matching time (Fig. 4b).

**Fig. 5:**
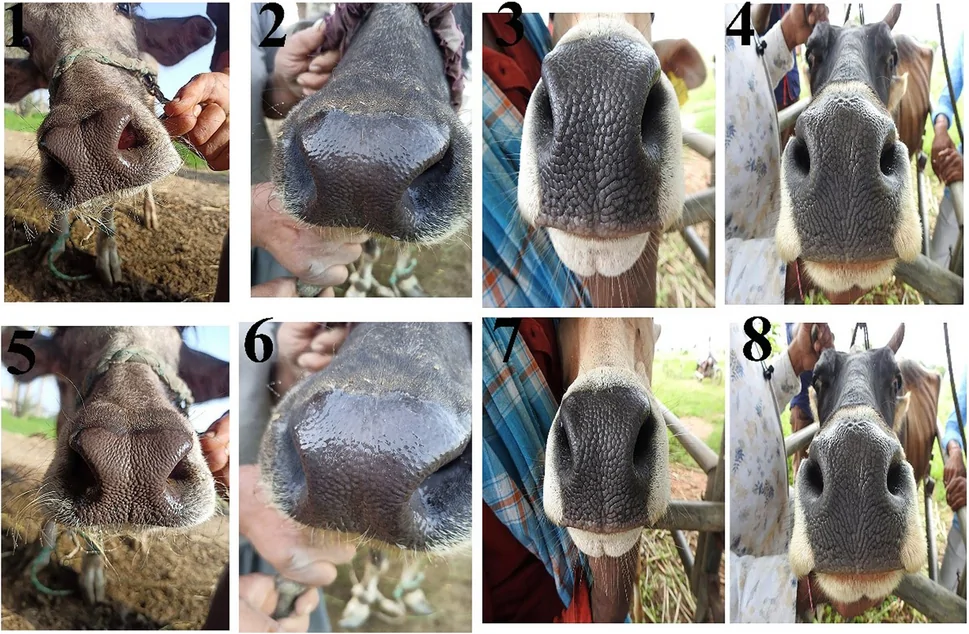
Examples of successful muzzle identification. The upper images represent enrolled templates (1 and 2, field cattle; 3 and 4, Kaggle dataset), while the lower images are query muzzle images that were successfully identified during testing (5,6 field cattle and 7,8 Kaggle dataset)

Figure 5 shows several identification examples. In all cases, the correctly identified animal was ranked first among the candidates with a similarity score above the decision threshold. The upper images represent enrolled templates (1 and 2, field animals; 3 and 4, Kaggle dataset), while the lower images are query muzzle images that were successfully identified during testing (5,6 field animals and 7,8 Kaggle dataset).

### Validation results

The model was validated using muzzle images from Kaggle dataset. The model recorded an accuracy of 90.3%, recall of 90.3%, precision of 95.37%, and an F1-score of 92.77% with a false rejection rate (FRR) of 9.78%. The similarity score was 88.42% (85.69,91.16). The images consumed about 36.91 (35.49,38.33) ms for feature extraction and the model took 5.9 (6.68,7.20) ms for matching the query image with the templates as shown in (Table 3).

**Table 3:** YOLOv8 configuration

Setting	Value
Initialization	`yolov8n.pt` pretrained weights
Task/classes	detection; one class, `muzzle`
Input	640 × 640
Epochs	50
Batch	8
Optimizer	AdamW
Learning rate	`0.001`, final factor `0.01`
Weight decay	`0.0005`
Warm-up	3 epochs
Schedule	cosine learning-rate schedule
Augmentation	mosaic 1.0, mixup 0.1, HSV, rotation 10°, translation 0.1, scale 0.5, horizontal flip 0.5
Seed/device	42 / CPU
Per-image confidence threshold	0.25
Detection/reference matching IoU	0.50

### Testing results

The performance of the model was evaluated using Kaggle dataset and field images. The model recorded an average cosine similarity of 88.44% with 95%CI (87.19,89.69) for Kaggle database and 86.55% (82.43,89.93) for field images. Also, the model achieved an average matching time 33.45 (33.98,36.59) ms for Kaggle dataset and 28.65 (24.18,32.29) ms for field images. The average feature extraction time was 106.53 (101.66,111,35) ms for Kaggle dataset, while field images consumed a longer extraction time of 2322.55 (2062.75,2510.89) ms as shown in Table 4. Stage-by-stage latencies were shown in Table 5; blur quality check consumed about 150–300 ms for field images and 2–5 ms for Kaggle ones. Muzzle detection required 1,500–1,800 ms and 65–75 ms for field images and Kaggle respectively. Field images took 150–300 ms for deep embedding and Kaggle images took 15–25 ms.

**Table 4:** Testing performance metrics of muzzle identification model using kaggle data base and field images

Dataset	Average cosine similarity (%),95%CI	Average matching time (ms), 95%CI	Average extraction time (ms), 95%CI
kaggle database	88.44 (87.19,89.69)	33.45 (33.98,36.59)	106.53 (101.66,111,35)
field images	86.55 (82.43,89.93)	28.65 (24.18,32.29)	2322.55 (2062.75,2510.89)

*95%CI* 95% confidence interval

**Table 5:** Stage-by-stage latencies

Pipeline Stage	Kaggle images	Field images
1. I/O & Decoding	~ 5–10 ms	~ 100–200 ms
2. Blur Quality Check	~ 2–5 ms	~ 150–300 ms
3. Muzzle Detection	~ 65–75 ms	~ 1,500–1,800 ms
4. CLAHE & Resize	~ 5–10 ms	~ 80–150 ms
5. Deep Embedding	~ 15–25 ms	~ 150–300 ms
Total Extraction Time	~ 106 ms	~ 2,322 ms

The model was tested on both Kaggle dataset and field images. For Kaggle dataset images, the model showed an accuracy of 96.2%, recall of 96.25%, precision of 99.35%, and F1-score of 97.77%, with a false rejection rate (FRR) of 3.75%. For field muzzles, the system recorded an accuracy of 80.76%, recall of 84%, precision of 95.45%, and F1-score of 89.58%, with an FRR of 16% (Table 6).

**Table 6:** Testing identification precision, recall, accuracy, and F1-score of muzzle identification model using Kaggle database and field images

Dataset	Accuracy (%)	Recall (%)	Precision (%)	F1-score (%)	FRR (%)
kaggle database	96.2	96.25	99.35	97.77	3.75
field images	80.76	84	95.45	89.58	16

In addition to evaluation on registered cattle, rejection performance was evaluated using unregistered cattle. The reported rejection rate was 97.5% and the reported false/incorrect acceptance rate was 2.5% as shown in Table 7.

**Table 7:** Rejection performance of the model on unregistered cows

Metric	Value (%)
Positive rejection rate (PRR)	97.5
False acceptance rate (FAR)	2.5

*PRR* True negative / Total impostor attempts *100

*FAR* FP / Total impostor attempts *100

Un registered probes = 80

The confusion information was reported in Table 8. Table 9 demonstrated the comparison of the study model with previous muzzle identification approaches.

**Table 8:** Confusion information

	Validation	Testing (kaggle database	Testing (field images)
Samples	320(2images per animal)	160 (one image per animal)	52 (one image per animal)
TP	288	154	42
FP	14	1	2
FN	31	6	8

*TP* True Positives, *FP* False Positives, *FN* False Negative

**Table 9:** Comparison of the study model with previous muzzle identification approaches

	Shojaeipour, Falzon et al. [27]	Li, Erickson et al. [29]	Digambar Pathak and Prakash [15]	Present study
Datasets	2900 images (beef, mixed breeds)	4,923 images	Dataset from Shojaeipour et al. [27]	kaggle database (1300 images) and 400 smartphone captured field images
animal counts	300	268	300	212
split strategy	5 shot/animal	65% training 15%validation 20% testing	57.0% training 21.6% validation 21.4% testing	Gallery set, Validation Set (320 images), Test Set (160 kaggle images and 52 field images)
Detector	YOLOv3	deep learning models (59 models)	YOLOv8	YOLOv8
embedding/classifier	ResNet-50+ transfer learning	14 image classification model families	EfficientNetV2B0	Efficient NetV2-S + ArcFace, 512-dimensional embedding spaces + cosine similarity
open-set handling	closed-set	closed-set	closed-set	closed-set (threshold based rejection rate and Impostor Rejection)
rank-1 performance	99.11%	98.7%	99.47%	96.2% for Kaggle database and 80.76% for field images.
field validation	Independent field validation was not reported	Images were captured from feedlots	Depend on public dataset	Preliminary field validation
model size	Not reported	Not specified	Not specified	81.92 MB
Latency	Not reported	28.3 ms	Not reported	33.45 ms for kaggle database and 28.65 for field images
FAR (unregistered cows)	Not reported	Not reported	Not reported	2.5%

## Discussion

The muzzle identification model was evaluated during the validation and testing stages to estimate its reliability under different conditions. The validation stage used the Kaggle dataset. The model recorded an accuracy of 90.3%, recall of 90.3%, precision of 95.37%, and an F1-score of 92.77%. These results indicated that the model was able to extract the muzzle features and correctly match cattle muzzle images during validation. During validation, the pipeline reported EER of 23.95%. in cases such as insurance claims, verifying a specific animal, or disease control, a false match animal has a high cost, the model utilizes a False Match Rate (FMR) ≤ 0.01. In routine management tasks, where the minimizing false rejections is critical, the model utilizes a False Non-Match Rate (FNMR) ≤ 0.05.

In the testing phase, the model showed shorter processing times and higher recognition performance on the Kaggle images than on the field images may be associated with higher image quality, controlled acquisition, and clear patterns. Field images presented a 1.87× larger pixel area compared to Kaggle images. The input tensor grew with this larger grid, consequently the YOLOv8 network had to search over a bigger feature map and expanding bounding-box regression before sending the cropped array to ArcFace. The reduction in similarity score in field images may be due to several factors, such as angle of the image, distance of the camera, variation in lighting, animal movement, and muzzle region distortion. These interpretations are plausible hypotheses due to lack of metadata. Also, the increased feature extraction time may reflect differences in image size, preprocessing load, or acquisition complexity.

The model showed differences in the performance in the level of accuracy, recall, precision, and F1-score that more observed in Kaggle database images than the field images. This difference may be due to higher quality of the Kaggle images, which may allow clear extraction of the muzzle features. The precision value indicates that most positive accepted cases corresponded to the true templates. The F1-score value indicated a balance between precision and recall. The observed value of FRR indicates that some genuine muzzle images were rejected, which may be due to variation in quality, orientation, and acquisition.

Registered cattle were used to evaluate the model’s capacity to identify the animal and unregistered ones to estimate the rejection performance. The model achieved a positive rejection rate for unregistered cows, indicating the application of the model for both rejection and identification scenarios.

The muzzle image was accepted as a match when the cosine similarity exceeded 0.70. Many query muzzles had similarity scores of 0.70 or higher but were rejected by the additional margin rule. Muzzle similarities between different animals may be greater than zero; therefore, the decision rule should be derived from empirical genuine and impostor score distributions. The second critical threshold that differentiates between these coincidental similarities and correctly rejects them is the angular margin threshold (a 3% margin threshold in the proposed decision rule).

Evaluating our model with previous literatures, our results that based on Kaggle dataset were in align with the results reported by Li et al. [29] but the preliminary field testing reported 15.44%-point absolute decrease in the accuracy below Kaggle test accuracy of 96.2%. Ruchay et al. reviewed that variations in animal posture, illumination, image acquisition protocols, and background clutter represent a challenge in precision livestock farming [43]. Ruchay et al. [44] proved that data augmentation can bridge this gap. Our model requires perspective normalization and sufficient augmentation for a production-ready application. For field validation, the accuracy must be ≥ 90% that reflects the minimum acceptable accuracy for herd management where 10% misidentification occurs, which will be controlled by secondary verification, the latency is less than 500 ms per image which will be suitable for scanning cattle in commercial feedlot during routine management and reduced the stress of forcing the animal to stand for an extended period, and the robustness less than 5% so that farm staff will trust the results of the system.

### Study limitations and future work

The model’s performance was not evaluated across different sub-populations or under varied lighting conditions, alternative capturing distances, motion blur, off-angle poses, reduced resolution or differing camera sensor. The results lack the long-term biometric stability. Future work should expand the dataset and evaluate robustness under a broader range of environmental conditions, optimize the model for inference on resource-constrained edge devices, validate the performance against pre-defined acceptance conditions, and focus on collecting stratify metadata on image quality covariates. Only after such demanding testing can the model be considered for operational deployment on-farm settings.

## Conclusion

This study proposed a Python-based deep learning biometric identification system for bovine using muzzle images. EfficientNetV2-S architecture was integrated with ArcFace (angular margin loss) for muzzle pattern extraction. The model was evaluated under controlled condition and certain field condition. Transitioning to field introduced domain-shift problem. Moving from the controlled to smart phone acquired field images reported absolute decrease of 15.44% points in the accuracy and about 20-fold increase in the feature-extraction time. These findings were strictly limited to certain condition and indicated that variations such as farms, breeds, devices, seasons, contamination states affected the model performance. The model evaluation remains insufficient for generalized field-readiness. Furthermore, the model can extract and match the query muzzles entirely offline, enhancing the practicality in remote farms where internet connectivity is limited. Overall, the model is a promising prototype for cattle identification.

## Supplementary Information

Supplementary Material 1.

## Data Availability

All data generated or analyzed during this study are available from the corresponding author upon reasonable request.

## Abbreviations

CLAHE: Contrast Limited Adaptive Histogram Equalization
CNN: Convolutional Neural Network
FRR: false rejection rate
YOLOv8: You Only Look Once
